# The Identification of Beckwith-Wiedemann Syndrome Through Swap Disentangled Variational Autoencoder

**DOI:** 10.1097/SCS.0000000000012540

**Published:** 2026-03-10

**Authors:** Tia Rijlaarsdam, Luke Smith, Alexander Rickart, Silvia Schievano, David Dunaway, Eppo Wolvius, Lara van de Lande, Simone Foti, Juling Ong

**Affiliations:** * University College London (UCL) Great Ormond Street Institute of Child Health, London, UK; † Erasmus Medical Center, Rotterdam, Netherlands; ‡ Craniofacial Unit, Great Ormond Street Hospital London, London, UK; § Department of Oral and Maxillofacial Surgery, Erasmus Medical Center, Rotterdam, Netherlands; ‖ Wellcome/EPSRC Centre for Interventional and Surgical Sciences, University College London; ¶ Department of Computing, Imperial College London, London, UK

**Keywords:** 3D stereophotogrammetry, artificial intelligence, autoencoders, Beckwith-Wiedemann syndrome, deep learning

## Abstract

Congenital syndromes with subtle changes in maxillofacial morphology can pose significant diagnostic challenges, wherein artificial intelligence holds great promise in aiding diagnosis through shape analysis. The authors applied the recently proposed Swap Disentangled Variational Autoencoder (SD-VAE) in diagnosis of Beckwith-Wiedemann syndrome (BWS). The SD-VAE model was trained on a data set primarily comprised of surface 3D head scans [stereophotogrammetry (3D SPG)], gathered using a 3dMD Head System (3dMD LLC). It was also trained on CT scans when available. A total of 72 syndromic scans were used belonging to 56 different BWS patients. Scans of head shapes were pre-processed and annotated with 68 anatomic landmarks. This aided in achieving uniformity between the scans and a template mesh, making a better comparison possible. The SD-VAE model outputs were then visualized in a 2-dimensional space and classified as ‘BWS-patient’ or ‘control’. For each anatomic facial region, the performance of the classification model was evaluated. This allowed us to understand the classification accuracy for each anatomic region as well as calculate the sensitivity and specificity for each region. The model demonstrated perfect diagnostic accuracy for BWS on the test set, with the most characteristic regions being the chin, cheeks, zygoma, eyes, jaw, and supraorbital region. This paper demonstrates how SD-VAE can be applied to 3D head meshes, to quantify the characteristic features of BWS. The authors distinguished BWS-specific features from those of the general population with high diagnostic accuracy. This makes SD-VAE a promising tool for aiding the referral and diagnosis of BWS in the future.

Diagnosis of many congenital disorders is often delayed owing to the rarity of the disease and the occurrence of mild or atypical cases. Early diagnosis is preferred, as it may have implications on clinical management. This is the case in Beckwith-Wiedemann syndrome (BWS), an overgrowth disorder with a prevalence of 1:10.000 caused by imprinting defects within the chromosome 11p15.5 segment.^1^ Several (epi)genetic alterations are known to cause BWS, namely: loss of function at CDKN1C, loss of methylation at imprinting center 2 (IC2LOM), gain of methylation at imprinting center 1 (IC1GOM), and (mosaic) segmental paternal uniparental isodisomy of chromosome 11p15 ((m)UPD11p15).^2^ The BWS phenotype is diverse and features include lateralized overgrowth, infraorbital creases, midfacial hypoplasia and most notably macroglossia (present in 90% of the patients^2^), with the resultant dentofacial complications of an anterior open bite, widened dental arches and prognathism.^2–4^ Tongue reduction surgery is frequently performed to improve speech, swallowing, occlusion and aesthetics, decrease drooling and relieve airway obstruction.^5,6^ With growth, the characteristic facial appearance of BWS tends to become milder. Because of the rarity of the disease, mild or atypical cases of BWS may get diagnosed later in life or go undiagnosed.^3,7^

BWS patients may have their diagnosis confirmed by molecular testing or be clinically diagnosed according to a scoring system defined by international consensus statement.^2^ This system considers cardinal features (eg, macroglossia) and suggestive features (such as ear pits and creases). Antenatally, a diagnosis of BWS may be suspected when ultrasonography (US) or magnetic resonance imaging (MRI) shows potential features of BWS.^8,9^ Postnatally, multimodal craniofacial imaging does not currently play a part in the diagnosis of this syndrome.

Early diagnosis of BWS allows for timely genetic testing, which can inform the need for counseling and appropriate screening. This is especially relevant considering several BWS genotypes are predisposed to embryonal tumor development in childhood, such as Wilms tumor and hepatoblastoma.^2^

Generally, tongue reductions performed before 3 years of age are preferred to achieve better outcomes in sleep apnea and potentially minimize the need for orthognathic surgery later in life.^10,11^ At Great Ormond Street Hospital (GOSH), we deem the optimal window for glossectomy to be between 1 to 2 years of age to optimize speech and dentofacial development.

Analysis of facial anatomy has evolved from 2D morphometrics to more advanced 3D techniques, largely aided by machine learning (ML). 3D facial shape data is high-dimensional, often comprising thousands of coordinates, prompting the use of dimensionality reduction methods like principal component analysis (PCA) and statistical shape analysis (SSA) in ML workflows. These are followed by classification or other ML approaches for shape analysis, proving able to diagnose craniofacial syndromes with high accuracy.^12–16^ Neural network (NN)-based models, such as variational autoencoders (VAEs), can extract low-dimensional shape representations (latent vectors), enabling the study of variation from a mean facial shape. A more refined model, the Swap Disentangled VAE (SD-VAE), enhances interpretability by corresponding a latent vector to a specific facial region. This builds upon earlier research, such as the work by O’Sullivan et al,^14^ allowing for more targeted analysis of regional shape contributions to the phenotype and postoperative facial changes.^17,18^

Given the challenges of early diagnosis and importance for appropriate treatment, as well as the characteristic features of BWS, we aimed to enhance our understanding of BWS facial features on a more granular level using ML techniques. Our aim was to analyze in which way BWS faces may be differentiated from the general population, to identify the contribution of each facial feature to the BWS phenotype, to find phenotype-genotype correlations and to assess postoperative facial changes.

## MATERIALS AND METHODS

### Data Sources

Patients diagnosed with BWS between 2007 and 2022 at Great Ormond Street Hospital for Children (GOSH), London, UK were included when they had routinely taken high-quality head scans [CT scans or 3D stereophotogrammetry scans (3D SPG)] and signed informed consent. Ethical approval was obtained from both the UK Research Ethics Committee (UK REC 15/LO/0386) and GOSH (R&D 12DS25). The resulting cohort will be referred to as “BWS-patient scans”. For the control cohort, CT-head scans of healthy children aged 0 to 4 years from Necker Children’s Hospital Paris, France and 3D SPG scans of the cranium of healthy children aged 4 to 17 from the Liverpool-York head model (LYHM),^19^ pre-processed by O’Sullivan et al.^14^ Given the rarity of BWS, data scarcity was compensated by performing a spectral data augmentation, which creates new meshes by random ‘’blending” (interpolation) of the spectral components from 2 meshes of the same group (BWS patient or control scans) and within the same age-range (0–4 y or >4 y).^17^ Groups with less data were augmented more heavily, to compensate for the significant class imbalance. Previous research showed that this technique plausibly combines features of the 2 real meshes, making the augmented meshes statistically suitable for data analysis.^17^

### Data Preprocessing

The included BWS-patient 3D SPG scans were imported into Materialise 3-matic V17.0 (Materialise NV) for manual preprocessing, which included bridging small holes in the mesh and fixing mesh discontinuities. 3D SPG scans with large parts missing or artefacts located in the face mesh and CT scans with a low number of slices were excluded. Most of the patients were wearing a hairnet during the 3D SPG scan. Surface irregularities caused by hair under the net had to be smoothed. Ears were removed as these are often insufficiently captured by 3D SPG and therefore cause registration issues. Any visible teeth or protruding tongues were manually removed to ensure a good registration. DICOM files of the included CT-head scans were imported into Materialise Mimics V25.0 (Materialise NV) and segmented using threshold tools for soft tissue. Artefacts such as tubes and cushioning were removed. The remaining face scan was then converted into a surface mesh.

### Scan Alignment

Meshes were put into dense correspondence with the LSFM^20^ template mesh to be further processed. This entailed the BWS-patient meshes being re-parameterized so each scan would exhibit the same topology, with the same number of vertices joined in a predefined triangulation and with the anatomic meaning of each point shared with the template mesh or across the cohorts.

A total of 68 landmarks were manually placed on each BWS-patient mesh, after the ibug68 facial landmarking convention.^21^ These landmarks were initially used to undertake Procrustes alignment; guiding rotation, translation, and scaling of the meshes to match the position and size of the template. Dense point correspondence was then achieved through landmark-guided non-rigid iterative closest point registration (NICP).^22^ This is a technique that adjusts each mesh so that the similar surface points (eg, outer corner of the lip) match up as closely as possible between meshes. Additional weighting values were defined to control the global and local stiffness of the template during the fitting procedure, ensuring the face-fitting process was accurate. This procedure was coded in Python and mostly relied on the Menpo3D library. When extra flexibility was needed to improve the fit of the facial template to a scan, additional adjustment was realized using Gaussian Processes (GP). This is a technique that allows smooth and controlled deformations of the template, especially in areas where more subtle or complex changes are required. GP was performed using the ‘’Scalismo” (Scalable Image Analysis and Shape Modeling) software library (https://scalismo.org/). The fitting accuracy of the deformed meshes compared with the pre-processed mesh was measured in Materialise 3-matic as the Hausdorff distance.

### Data Analysis

Once dense correspondence was achieved for all the meshes, they were compressed using a mesh VAE. VAE are neural networks that learn a mapping between shapes and a low-dimensional numerical representation by using an encoder-decoder architecture. As the name suggests, the encoder encodes input data into normally distributed latent vectors, the decoder generates new shapes from such vectors.

In this paper, we applied SD-VAE, which is capable of creating more interpretable, structured, and disentangled feature vectors than previous VAEs. We trained SD-VAE to disentangle 70 latent variables into 14 subsets of 5 variables. Subsets of variables within each vector represented different anatomic sub-units of the head (eg, nose, chin, cheeks).^17,23^

The model was trained for 800 epochs using a batch size of 16 with a learning rate of 1*e* ^−4^.

Data were divided into 3 sets: a training, validation and a test set. The stratified split was manually adjusted to fulfill 3 requirements (i) the training set must contain as many genotypes as possible; (ii) the validation set must contain as many clinical diagnoses as possible, as these cannot be used for genotype classification; (iii) the test set must contain patients with both preoperative and postoperative scans for comparison.

### Data Classification

The classification of facial regions was performed through Quadratic Discriminant Analysis (QDA), a statistical method that analyzes examples from the training data set and learns statistical patterns in the data. QDA assumes that each region follows its own (Gaussian) distribution and once trained, can look at new and unseen meshes from the “test” set and assign which group a shape belongs to. The latent vectors of SD-VAE were classified both on a global and local level as being either BWS or healthy. One QDA classifier was trained to operate on the whole head, and an additional classifier was trained for each subset of latent variables representing a different anatomic region. For each region, a confusion matrix was created to evaluate the performance of the classification model. It compares the true labels (BWS or control) with the predicted labels (which the model guessed). This allows us to understand the classification accuracy for each anatomic region, as well as calculate the sensitivity and specificity for each region.

**FIGURE 1:**
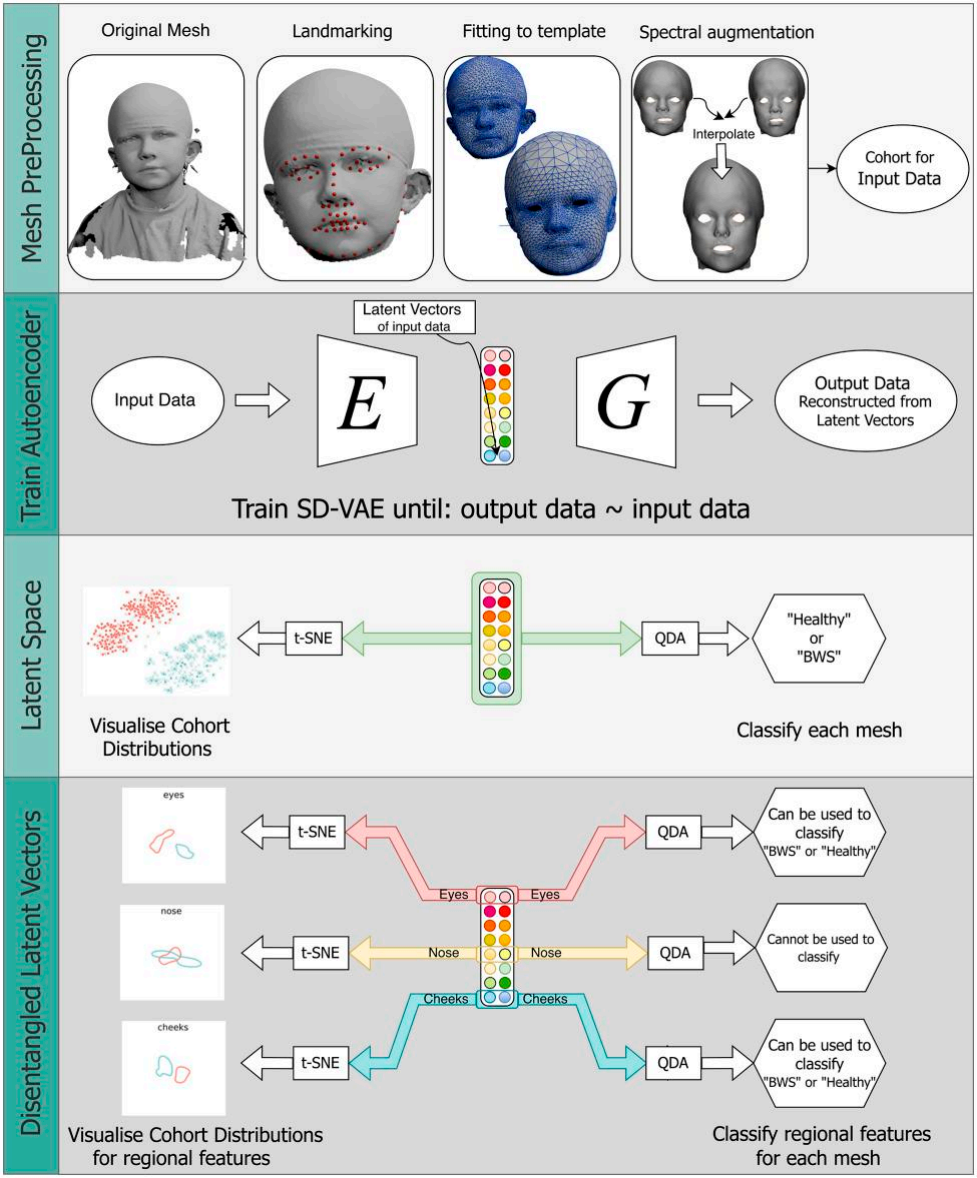
Flowchart visualizing mesh formation, mesh refinement and alignment, latent vector encoding, and classification and representation using the Swap Disentangled Variational Autoencoder (SD-VAE). For classification and representation t-Distributed Stochastic Neighbour Embedding (t-SNE) and Quadratic Discriminant Analysis (QDA) methods were applied.

To demonstrate results, each facial region’s latent vector was plotted into a 2-dimensional space through t-Distributed Stochastic Neighbour Embedding (t-SNE). This allowed us to quantify the overlap (and thus facial similarities) between BWS patients and those in the control group. Age at scan and sex were also accounted for. The distributions of different genotypes were overlaid on top of the BWS and control populations to visualize potential genotype-phenotype correlations. For patients who had undergone tongue reduction surgery, and there were appropriate scans, it was also possible to plot the preoperative and postoperative latent representations, again using t-SNE. This allows us to visualize how surgery affected the facial morphology with respect to not only the healthy population but also the unoperated phenotype. The methods described above have been visualized in a flowchart (Fig. 1).

## RESULTS

Seventy-two patients met the inclusion criteria (supplementary table 1, Supplemental Digital Content 4, http://links.lww.com/SCS/J138) with IC2LOM being the most prevalent genotype (supplementary table 2, Supplemental Digital Content 5, http://links.lww.com/SCS/J139). Of these 72, 69 (95.8%) were obtained using 3D SPG, and 3 (4.2%) were obtained by CT. 3D SPG scans are taken using the 3dMD Head System (3dMD LLC). After performing the stratified split, 2 of the total 27 pre-op BWS-patient scans were placed in the validation set and 3 in the test set. These meshes were not augmented to assess the capabilities of our model only on real patients. The remaining 22 pre-op scans were augmented and used in the training set.

### Dense Correspondence and Data Augmentation

For all 72 scans, the median Euclidean distance between each point on the registered NICP/GP mesh and the corresponding pre-processed patient mesh was <1 mm. The data was augmented by a factor of 5, with the heaviest augmentation being the BWS meshes. Several examples of augmented BWS-patient scans can be seen in supplemental digital content 1, http://links.lww.com/SCS/J135 showing these scans are comparable to ‘real’ face scans.

In supplemental digital content 2, http://links.lww.com/SCS/J136 it can be seen that augmentation is effective, as it fills in regions within the BWS distribution which would otherwise be empty due to data scarcity.

### Preoperative Global Latent Distribution

**FIGURE 2:**
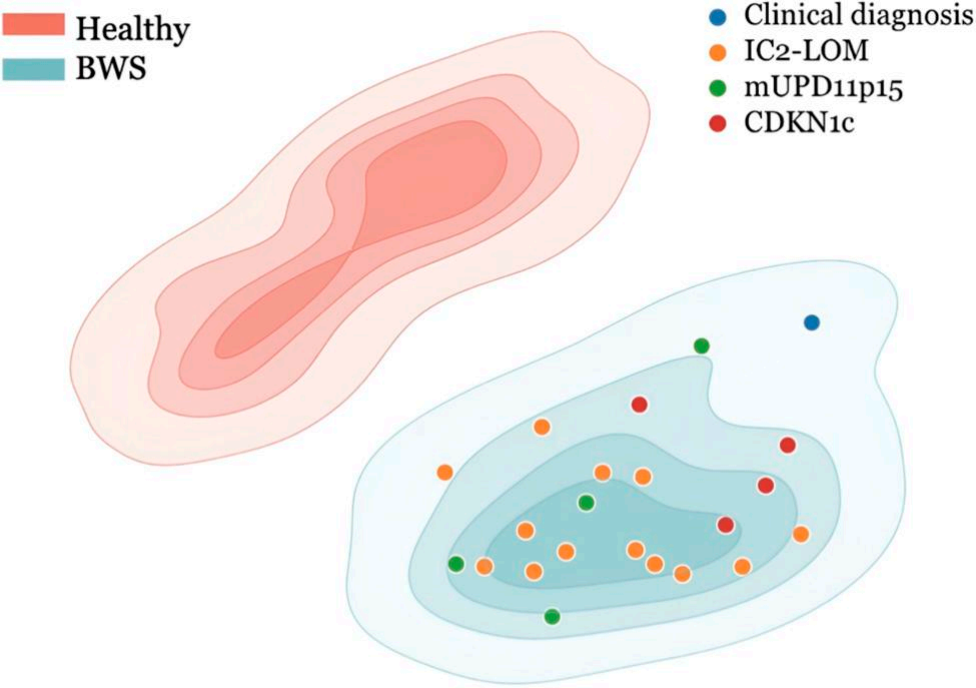
Latent space visualization of BWS-patient genotypes and one clinically diagnosed patient, overlaid over the healthy versus BWS-patient distributions.

Given the global latent representations computed with SD-VAE for all subjects, QDA reported a 100% test accuracy. Similarly, t-SNE showed 2 well-separated distributions of the 2 study populations, that is, healthy and BWS (see supplemental digital content 3, http://links.lww.com/SCS/J137). Latent space visualization of the BWS-patient genotypes did not demonstrate any clustering based on phenotype, age, or sex (Fig. 2).

### Postoperative and Anatomic Region Latent Distribution

**FIGURE 3:**
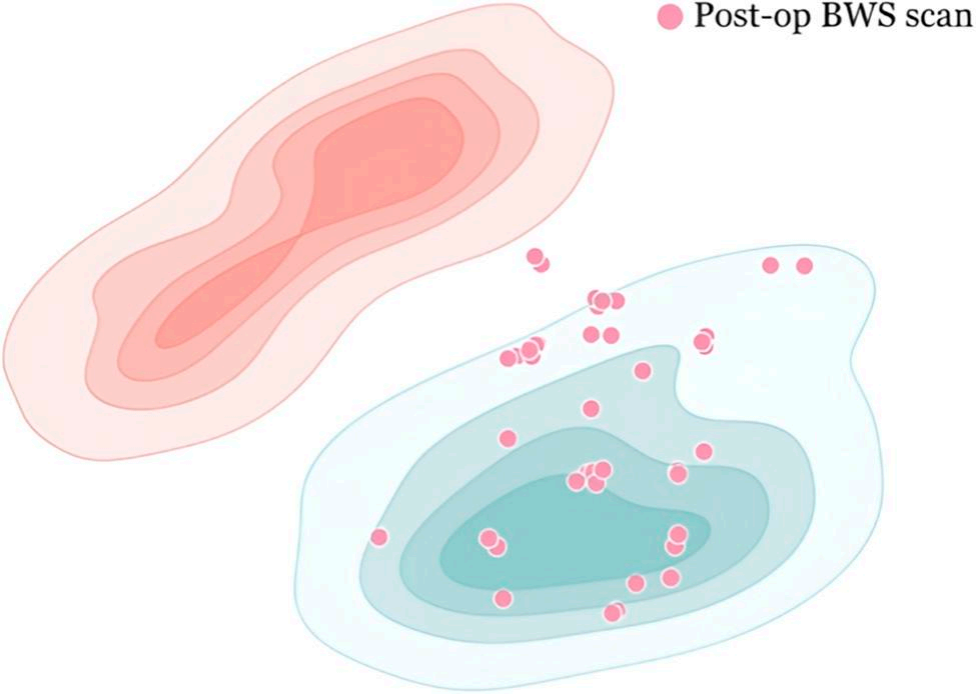
Latent space visualization of the post-op BWS-patient scans, overlaid over the healthy versus pre-op distributions.

Postoperatively, the patients seem to cluster more towards the outer layers of the BWS group, however, do not reach the distribution of healthy populations, suggesting that after tongue reduction, patients remain different from the healthy population (Fig. 3).

**FIGURE 4:**
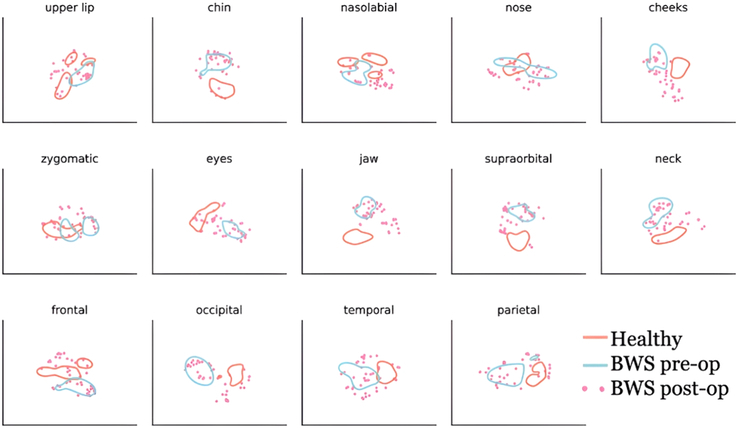
Disentanglement of the anatomic region latents of the healthy versus BWS group. The disentangled latents of post-op BWS meshes are overlaid. At the top of each plot, the disentangled region is defined. When the healthy and BWS-patient distributions do not overlap, SD-VAE suggests that the specific anatomic region contributes to the characterization of the syndrome. When a cluster splits into two (or more), this is because data from that class tends to cluster in very specific, yet distant, parts of the latent space.

Figure 4 depicts the t-SNE embeddings of the 14 subsets of latent variables disentangled by SD-VAE and corresponding to the different anatomic regions, as well as the BWS postoperative scans scattered on top of the data distributions. Except for the nose and zygomatic region, the 2 classes (ie, “BWS pre-op” and “control”) are separated in the latent spaces, suggesting that all but these 2 facial regions differ between BWS patients and the control group. In addition, the post-op scans, which are scattered onto the distributions of Figure 4, tend to exhibit an appearance trending towards the healthy or unaffected population in most regions.

**FIGURE 5:**
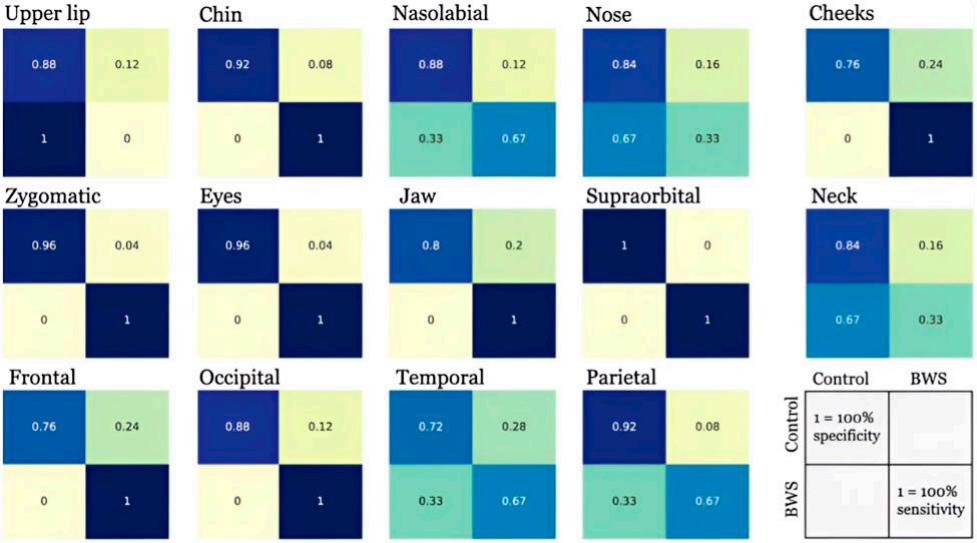
Confusion matrices for each disentangled anatomic region in the test set. For each square, the top left quadrant corresponds to specificity and the lower right quadrant corresponds to sensitivity. The remaining 2 quadrants measure the misclassification. 1=100%. *x* axis = true label, *y* axis = predicted label.

Figure 5 shows the corresponding class separability per facial region is also quantified, which depicts the confusion matrices evaluating the classification performances of the region-specific classifiers in only the test set. This shows us the region with the highest assignment accuracy achieved by SD-VAE in the test set is the supraorbital region, closely followed by the zygoma, eyes, and chin.

## DISCUSSION

### Preoperative Appearance

By using SD-VAE, we were able to quantify the effect of each anatomic subunit on the facial appearance of BWS patients. Many of the characteristic anatomic regions identified by SD-VAE can be linked to the typical regions previously described in the literature. For instance, the distinguishable eyes, cheeks, and jaw regions can be linked to previously reported infraorbital creases, midface hypoplasia, and prognathism, respectively.^2–4^ Looking at Figure 4, SD-VAE finds the upper lip and nasolabial folds to be mostly characteristic for BWS. Several other regions identified by SD-VAE as being distinguishable for BWS, have not previously been reported in the literature to the authors’ knowledge. These include the supraorbital region, occipital region, and parietal region. The differences observed in the occipital and parietal regions may be influenced by variations in hair smoothing during facial scan preprocessing. However, the supraorbital region was not manually pre-processed and may form a new region of importance within the facial morphology of BWS and can thus aid physicians and AI-tools in their clinical recognition of the disease. This claim is supported by the perfect classification accuracy in the corresponding confusion matrix (Fig. 5).

When addressing the facial morphology, this research found no significant differences among the different BWS genotypes within the current data set. In this analysis, there is a mild trend in IC2LOM and CDKN1c clustering, which is not statistically significant. Notably, Defabianis and colleagues reported significant correlations between skeletal jaw relationships and molecular subtypes, with mUPD11p15 patients displaying class I and clinically diagnosed patients more frequently displaying class II relationships. Although they reported differing trends in maxillofacial growth potential, their study included no patients with the CDKN1 genotype.^4^ Mussa et al^24^ reported macroglossia being present significantly less often in mUPD11p15 patients. Both these findings suggest there being genotype-phenotype differences present in BWS. The lack of statistical significance in our study is likely caused by the limited amount of input data available owing to the rarity of the disease and the imbalance in the genotype subgroups. Regardless, it is certainly possible for SD-VAE to identify genotype-phenotype correlations and this represents an avenue for future research.

### Postoperative Appearance

Literature reports that the facial appearance of BWS tends to become milder with age and older patients who have undergone tongue reduction often do not look strikingly different from the general population.^3^ The results presented in this paper however suggest otherwise.^3^ As SD-VAE was trained using only unoperated BWS patients, this data set was composed of mostly young individuals. However, the patients who had undergone tongue reduction surgery and were therefore older, did not fully move into the normal distribution, highlighting that the morphological changes driven by macroglossia and overgrowth have a tendency to persist. In addition, this suggests SD-VAE is sensitive to subtle facial dysmorphism in BWS patients, making it a useful tool in the future for aiding clinicians in diagnosing milder cases of BWS. This claim is supported by previous findings of facial recognition tools having comparable or higher success rates than clinical professionals in the identification of rare diseases.^14,25^ In addition, with more available data, SD-VAE holds the potential to help predict the postoperative outcomes as well as the morphologic changes that may occur as during childhood and adolescence.

### Clinical Significance

Early diagnosis of BWS is crucial for optimal management, especially when considering appropriate tumor screening and timely intervention to drive better long-term outcomes.

SD-VAE holds potential for improving and aiding in the referral and diagnosis of not only BWS, but a wider subset of craniofacial syndromes. This is especially beneficial considering developments in automatic landmarking techniques,^26^ handheld 3D SPG scanners,^27^ and smartphone scanning, which have the ability to make provisional diagnosis a viable option in primary care or even low-resource environments.^28^ Similarly, it may help in cases where genetic testing is equivocal or declined, while also aiding in the referral of mild or atypical cases. It is also likely to be possible to translate this work to the antenatal setting, combining SD-VAE with 3D antenatal ultrasound or fetal MRI to facilitate the diagnosis of craniofacial anomalies in utero.

### Limitations

As with many rare diseases, the most relevant limitation of this paper is the scarcity of available data. Considering the small batch of BWS-patient scans used for testing, more data is needed to further develop the model. We would advocate for the routine capture of 3D photogrammetry at regular intervals throughout each patient’s journey, allowing us to catalogue facial growth and development over time. Although ambitious, we would also support national and international data-sharing of 3D SPG images for not only BWS patients but also the wider population of patients affected by craniofacial syndromes, which would provide a rich database for future research.

## CONCLUSIONS

This study applied SD-VAE to scans of patients with BWS to objectively identify and quantify the facial regions contributing to the phenotype. SD-VAE successfully diagnosed BWS across different 3D scan modalities while also highlighting the specific craniofacial regions that are most characteristic for the condition. By analyzing head scans, the model accurately classified craniofacial differences in pediatric BWS patients compared with the control group. Future work could focus on expanding SD-VAE’s application to other craniofacial syndromes and integrating it into clinical diagnostic processes.

## Supplementary Material
