# Supplementary material for: The Identification of Beckwith-Wiedemann Syndrome Through Swap Disentangled Variational Autoencoder
Source: J Craniofac Surg. 2026 Mar 10;37(7):1921–6. doi: 10.1097/SCS.0000000000012540 (PMC13290057; doi:10.1097/SCS.0000000000012540)

**Supplemental Digital Content 2** Latent space visualization of SD-VAE trained on real and augmented data. The augmented scans fill in otherwise empty space.


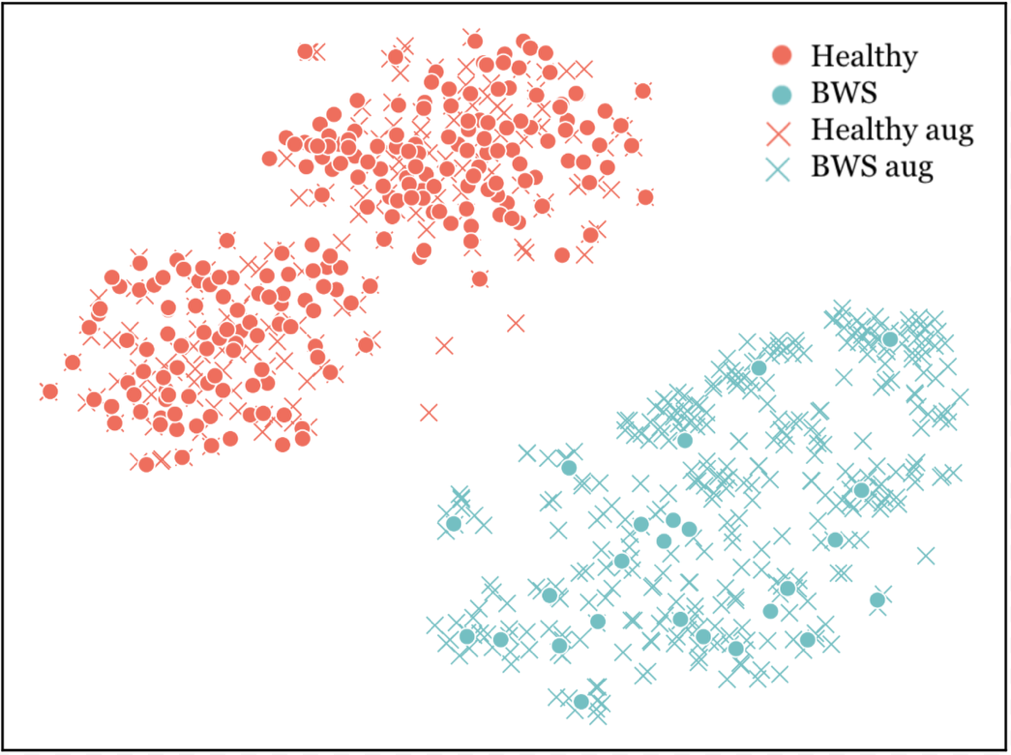

Supplement: Supplementary file 2 [file scs-37-1921-s002.docx]
