# Supplementary material for: The Identification of Beckwith-Wiedemann Syndrome Through Swap Disentangled Variational Autoencoder
Source: J Craniofac Surg. 2026 Mar 10;37(7):1921–6. doi: 10.1097/SCS.0000000000012540 (PMC13290057; doi:10.1097/SCS.0000000000012540)

**Supplemental Digital Content 3** Latent space visualization of the test set (consisting of BWS patients), compared to the train set (consisting of BWS patients).


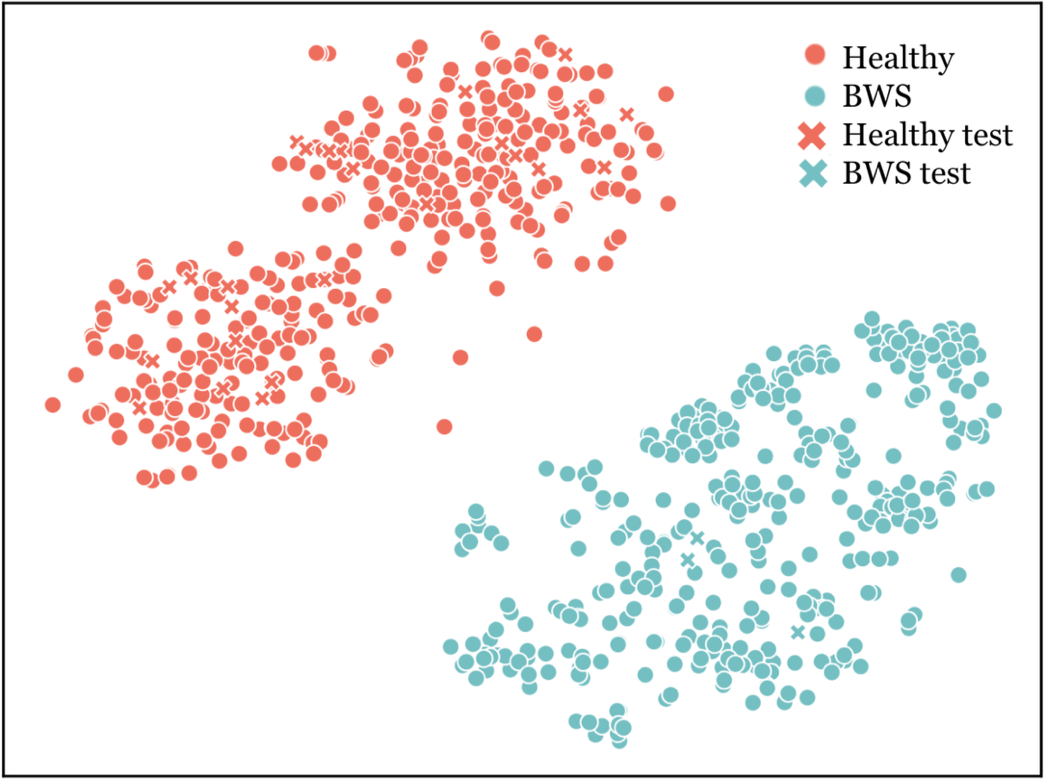

Supplement: Supplementary file 3 [file scs-37-1921-s003.docx]
