# Supplementary material for: The Identification of Beckwith-Wiedemann Syndrome Through Swap Disentangled Variational Autoencoder
Source: J Craniofac Surg. 2026 Mar 10;37(7):1921–6. doi: 10.1097/SCS.0000000000012540 (PMC13290057; doi:10.1097/SCS.0000000000012540)
Supplement: Supplementary file 4 [file scs-37-1921-s004.docx]

**Supplemental Table 1** Overview of BWS-patient scan dataset. The pre/post-op scan division alludes to the first glossectomy performed on the BWS patient.

| **Characteristic** | |
| --- | --- |
| Total BWS patients - no. | 56 |
| Total BWS-patient scans - no. | 72 |
| Sex, male - no. (%) | 24 (42.9) |
| Age at scan, median - years (IQR) | 3.0 (1y 5mo - 7y 3mo) |
| Age range at scan - years | 9mo - 17y 8mo |
| Total pre-op scans - no. (%) | 27 (37.5) |
| BWS patients with both pre- and post-op scans - no. (%) | 9 (16.1) |
