# Supplementary material for: The Identification of Beckwith-Wiedemann Syndrome Through Swap Disentangled Variational Autoencoder
Source: J Craniofac Surg. 2026 Mar 10;37(7):1921–6. doi: 10.1097/SCS.0000000000012540 (PMC13290057; doi:10.1097/SCS.0000000000012540)
Supplement: Supplementary file 5 [file scs-37-1921-s005.docx]

**Supplemental Table 2** Overview of the genotype prevalence of the total BWS-patient cohort and the genotype prevalence of only the pre-glossectomy scans.

| **Genotype** | **No. (%) of total patients** | **No. (%) of total pre-op scans** |
| --- | --- | --- |
| IC2-LOM | 38 (67.9) | 15 (55.6%) |
| mUPD11p15 | 11 (19.6) | 5 (18.5%) |
| CDKN1c | 4 (7.1) | 4 (14.8%) |
| Clinical diagnosis | 3 (5.4) | 3 (11.1%) |
